# Pharmacokinetics, mass balance, and metabolism of the novel potassium-competitive acid blocker JP-1366 in healthy Chinese adults following a single oral dose of [^14^C]JP-1366

**DOI:** 10.3389/fphar.2025.1701581

**Published:** 2025-11-10

**Authors:** Xuemei Liu, Decheng Deng, Jian Meng, Haitang Hu, Quankun Zhuang, Xue Zhou, Long Fu, Binke Fan, Xueting Xu, Qin Huang, Xiaoyan Chen, Fang Hou

**Affiliations:** 1 Phase I Clinical Research Center, Beijing GoBroad Hospital, Beijing, China; 2 Livzon Pharmaceutical Group Co., Ltd., Zhuhai, China; 3 Shanghai Institute of Materia Medica, Chinese Academy of Sciences, Shanghai, China

**Keywords:** JP-1366, P-CABs, mass balance study, radioactivity, drug metabolism, pharmacokinetics

## Abstract

**Introduction:**

JP-1366 (zastaprazan), a novel potassium-competitive acid blocker (P-CAB), was approved in South Korea in 2024 for treating erosive gastroesophageal reflux disease (GERD). This mass balance study characterized the pharmacokinetics, metabolism, excretion, and safety profile of JP-1366 in humans, supporting its further clinical development in China.

**Methods:**

Six healthy Chinese male adults received a single oral dose of 20 mg (100 μCi) [^14^C]JP-1366 under fasting conditions in this open-label Phase I study. Serial samples of blood, plasma, urine, and feces were collected and analyzed to determine Pharmacokinetic parameters, total radioactive recovery, metabolic fate, and excretion routes.

**Results:**

JP-1366 was rapidly absorbed, with median T_max_ values of 0.875 h observed for both the parent drug and total radioactivity in human plasma. The mean plasma elimination half-life (t_1/2_) was approximately 28 h for JP-1366-related material. Furthermore, the blood-to-plasma ratio of total radioactivity ranged from 0.561 to 0.645, indicating limited distribution of drug-related material into blood cells. Within 264 h post-dose, 94.3% of the administered radioactive dose was recovered through excretion, predominantly via feces (51.9%) and urine (42.4%). JP-1366 underwent extensive metabolism *in vivo*, generating 57 metabolites, while the parent drug remained undetectable in excreted samples. The principal metabolic pathways involved oxidations (Phase I) and the following glucuronidation (Phase I/II). JP-1366 exhibited a favorable safety profile, with no serious adverse events reported.

**Conclusion:**

JP-1366 exhibited favorable pharmacokinetic properties characterized by rapid absorption, extensive metabolism, and predominant fecal excretion of drug-related material. The principal metabolic pathways involved oxidations and the following glucuronidation.

**Clinical Trial Registration:**

http://www.chinadrugtrials.org.cn/, identifier CTR20244931.

## 1 Introduction

Gastroesophageal reflux disease (GERD), a chronic gastrointestinal disorder, is pathologically defined by the retrograde flow of gastric/duodenal contents into the esophagus, clinically manifesting as recurrent heartburn and regurgitation (Maret-Ouda et al., 2020). The estimated global prevalence among adults is 10%–20% with regional variability and demonstrates an increasing trend (Nirwan et al., 2020; Li et al., 2023; Wickramasinghe and Devanarayan, 2025). Current evidence indicates that genetic predisposition, environmental factors, psychological factors, and poor lifestyle/dietary habits contribute to its pathogenesis (Gong et al., 2024; Taraszewska, 2021; Yuan and Larsson, 2022; Zamani et al., 2023).

As a novel class of reversible H^+^/K^+^-ATPase inhibitors, potassium-competitive acid blockers (P-CABs) provide effective and sustained suppression of gastric acid secretion (Scarpignato and Hunt, 2024; Kasugai and Ogasawara, 2024; Tietto et al., 2025). Unlike conventional proton pump inhibitors (PPIs) requiring acid-mediated activation, P-CABs exhibit rapid onset of action and food intake-independent efficacy, offering a mechanistically distinct therapeutic option for acid-related disorders such as GERD. P-CABs inhibit both resting and activated proton pumps, achieving maximal acid suppression with a single dose without the need for repeated administration. In contrast, traditional PPIs require 3–5 days of continuous dosing to reach full therapeutic effect. The first P-CAB, Revaprazan, was approved in Korea in 2007 (Sugano, 2018). Currently marketed P-CAB drugs also include Vonoprazan (St Onge and Phillips, 2023), Tegoprazan (Wang et al., 2025; Mermelstein et al., 2020), Fexuprazan (Ramani et al., 2023), Keverprazan (Kang, 2023).

JP-1366 (Zastaprazan), a novel potassium-competitive acid blocker (P-CAB) developed by Onconic Therapeutics Inc. (Seoul, South Korea), demonstrates potent gastric acid-suppressing efficacy with a favorable safety profile (Ku et al., 2023; Hwang et al., 2023). JP-1366 was approved in South Korea in April 2024 for the treatment of erosive GERD at a once-daily dose of 20 mg (Yang et al., 2024). Its commercialization rights in Greater China (mainland China, Hong Kong, Macau, and Taiwan) were exclusively licensed to Livzon Pharmaceutical Group Co., Ltd. (Zhuhai, China) in March 2023. Following single-dose administration (5–60 mg) and multiple-dose administration (5–40 mg), JP-1366 exhibited dose-proportional increases in systemic exposure. For JP-1366, the time to peak (T_max_) plasma concentration was approximately 0.5–2.0 h, with a mean terminal elimination half-life (t_1/2_) of 6–10 h. In addition, *in vitro* studies suggested that JP-1366 was predominantly metabolized by cytochrome P450 3A4/5, and CYP2C19 phenotypes had no significant effect on exposure based on the population pharmacokinetic analysis of JP-1366 (Ku et al., 2023; Hwang et al., 2023; Yang et al., 2024). To date, human metabolism and excretion pathways remain uncharacterized, which compromises the evidence base for dosing recommendations in drug-drug interactions (DDI) and special populations.

To further elucidate the absorption, metabolism, and excretion characteristics of JP-1366 in humans, a mass balance study of [^14^C]JP-1366 in healthy Chinese adult male study participants was proposed. This study aims to reveal the pharmacokinetic characteristics of JP-1366 and provide a reference for its rational use in clinical practice.

## 2 Materials and methods

### 2.1 Study design

This was a single-center, open-label, non-randomized, single-dose study conducted in healthy Chinese male study participants, with a minimum of 6 study participants required to complete the trial. It comprised a screening period (D-28 to D-2), baseline period (D-2 to D-1), and treatment period (D1 to D8). Study participants received a single oral dose of 20 mg (100 µCi) [^14^C]JP-1366 on D1 and stayed in the clinical research unit for 8 days after dosing. Serial blood samples were collected at predefined time points from 0 to 168 h post-dose. A baseline urine sample and a fecal sample were obtained within 24 h prior to dosing, while complete urine and fecal collections occurred at scheduled intervals from 0 to 168 h post-dose. Sample collection could be prematurely terminated or extended depending on whether prespecified stopping criteria were met. Safety monitoring was performed throughout the study.

The study was approved by the Ethics Committee of Beijing Gobroad Hospital (No. DR 2024-029-001) and conducted in accordance with the Declaration of Helsinki and Good Clinical Practice (GCP) guidelines. The trial was registered at the Chinese Clinical Trial Registry (www.chinadrugtrials.org.cn, Registration No. CTR20244931).

### 2.2 Study participant selection and enrollment

All study participants provided written informed consent prior to enrollment, following a comprehensive explanation of the trial’s objectives, procedures, potential risks, and benefits. Key inclusion criteria included: (1) Healthy Chinese males aged 18–45 years; (2) Body mass index (BMI) 19–26 kg/m^2^; (3) Willingness to comply with all protocol-specified procedures. Main exclusion criteria included: (1) History of hypersensitivity, or known allergy to P-CABs, PPIs or other medications (e.g., aspirin, antimicrobial agents); (2) History of clinically significant diseases in major organ systems (e.g., neurological, endocrine, cardiovascular, respiratory, gastrointestinal, renal/urinary, or reproductive); (3) Clinically significant abnormalities in vital signs, physical examination, laboratory tests, 12-lead ECG, abdominal ultrasound or chest radiography; (4) Severe gastrointestinal conditions like hemorrhoids with active bleeding, severe nausea/vomiting, or chronic constipation/diarrhea; (5) Gastrointestinal surgery affecting drug absorption, or other factors affecting pharmacokinetics; (6) Significant radiation exposure within the past 12 months.

### 2.3 Drug formulation and administration

**FIGURE 1:**
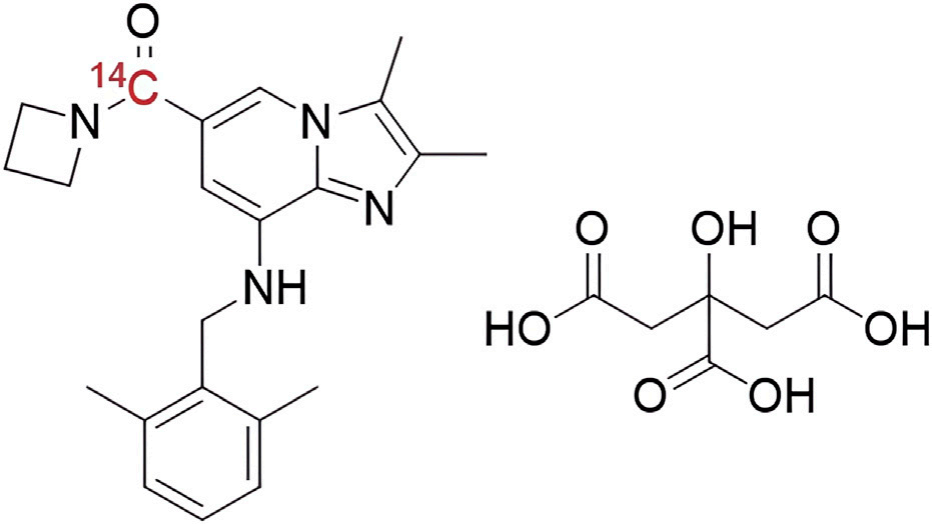
The chemical structure of [^14^C]JP-1366.

JP-1366 was provided by Livzon Pharmaceutical Group Co., Ltd. (Zhuhai, China), and the [^14^C]-labeled JP-1366 compound (specific activity: 52.39 mCi/mmol, radiochemical purity: 100%, chemical structure shown in Figure 1) was synthesized by Wuxi Beita Pharmatech Co., Ltd. (Wuxi, China). Prior to the trial, a suspension of [^14^C]JP-1366 (20 mg containing 100 µCi radioactivity) was prepared for each study participant in 60 mL of 0.5% (*w/v*) methylcellulose solution by the Shanghai Institute of Materia Medica. The formulation was stored at −20 °C and then transferred to a 2–8 °C refrigerator for thawing within 24 h prior to dosing.

Following a ≥10-h overnight fast, study participants received a single oral dose of 20 mg (100 µCi) [^14^C]JP-1366 suspension. Water intake was prohibited from 1 h pre-dose to 1 h post-dose, except for 240 mL administered with the drug. Fasting continued for 4 h post-dose, after which a standardized meal was provided.

### 2.4 Sample collection and handling

Blood samples were collected in K_2_-EDTA tubes pre-dose (within 60 min before dosing) and at 0.25, 0.5, 0.75, 1, 1.5, 2, 3, 4, 6, 8, 12, 24, 48, 72, 96, 120, and 168 h post-dose. Plasma samples were isolated by centrifugation (1700×g, 10 min, 4 °C) within 1.5 h of draw. All blood and plasma samples were stored at −80 °C until analysis. Based on predefined stopping criteria, sample collection was terminated at 168 h because the measured total radioactivity in the collected plasma was three times lower than that of the pre-dose (background level). Blank urine and fecal samples were collected within the 24 h period prior to dosing. Post-dose urine samples were collected at consecutive intervals: 0–4 h, 4–8 h, 8–24 h, 24–48 h, 48–72 h, 72–96 h, 96–120 h, 120–144 h, and 144–168 h. Fecal samples were collected during the following intervals: 0–24 h, 24–48 h, 48–72 h, 72–96 h, 96–120 h, 120–144 h, and 144–168 h. To prevent drug adsorption, freshly collected urine samples were supplemented with a 20% Tween-80 solution at a 100:1 ratio (*w/v*, urine:surfactant). Vomitus samples occurring post-administration were retained for potential bioanalysis. All excreta samples were stored at −80 °C pending analysis. Excreta collection was discontinued when both of the following conditions were satisfied: (1) Total excreted radioactivity exceeded 90% of the administered dose; (2) Cumulative excretion was <1% of the administered dose on two consecutive days. Based on the interim data, excreta collection was extended up to 216 h for study participant R001, R004, and R006, and up to 264 h for study participant R002, R003, and R005, with extensions implemented at 24 h intervals.

For stability assessment, plasma and urine samples were analyzed after being stored at room temperature for 23 h and 24 h, respectively. The observed deviations between the measured and theoretical values for both the parent drug M0 and the metabolite M379-1 (M1) were less than 10%. This confirms the stability of the drug in these matrices. Furthermore, fecal samples from each participant were immediately stored in an ultra-low temperature freezer at −70 °C after every bowel movement, thereby ensuring the stability of the drug in feces.

### 2.5 Sample analysis

#### 2.5.1 Radioactivity measurement (blood, plasma and excreta)

Radioactivity in blood, plasma, and excreta was quantified by liquid scintillation counting (LSC) using a Tri-Carb 3110 TR counter (PerkinElmer, Wellesley, MA, United States). For plasma and urine samples, an appropriate aliquot was transferred to a scintillation vial and weighed after vortex-mixing for 1 min. Subsequently, 20 mL of scintillation cocktail was added, and the mixture was vortex-mixed thoroughly before analysis. Fecal samples were homogenized with twice their weight of methanol-water (1:1, *v/v*). Aliquots of blood and fecal homogenate were precisely weighed, then combusted for 4 min in an OX-501 biological oxidizer (Harvey, NY, United States), and the liberated ^14^CO_2_ was trapped in 20 mL of scintillation cocktail for subsequent LSC analysis.

#### 2.5.2 Analysis of JP-1366 and its metabolite M1 in plasma

A robust and validated high-performance liquid chromatography coupled with tandem mass spectrometry (HPLC-MS/MS) method was developed for the determination of unlabeled JP-1366 and its metabolite M1 in plasma. JP-1366 (purity: 99.53%) and its metabolite M1 (purity: 96.1%) were quantitated using a common deuterated internal standard ([^3^H_6_]JP-1366; purity: 93.9%). These reference standards were provided by Livzon Pharmaceutical Group Co., Ltd. (Zhuhai, China). Plasma samples were pretreated via protein precipitation using acetonitrile. Chromatographic separation was achieved using an Agilent Eclipse Plus C18 column (4.6 × 100 mm, 3.5 µm) with a mobile phase consisting of (A) 5 mM ammonium acetate in water containing 0.2% formic acid and (B) acetonitrile. Detection was performed using a Triple Quad™ 5500 tandem mass spectrometer (SCIEX, Framingham, MA, United States) operating in positive electrospray ionization (ESI) mode with multiple reaction monitoring (MRM). The method was validated over the concentration range of 1.00–500 ng/mL for both analytes, with a lower limit of quantification (LLOQ) of 1.00 ng/mL.

#### 2.5.3 Metabolite profiling and identification

Based on PK data and excretion rate, plasma, urine, and fecal samples collected at various time points or intervals were pooled individually for comprehensive metabolite analysis. The plasma samples collected at different time points (0–48 h) from six study participants were pooled individually according to the Hamilton method (Hamilton et al., 1981; Hop et al., 1998). Urine was proportionally pooled by volume (0.2% per collection interval) to generate a composite urine sample for each study participant across 0–48 h. Feces was proportionally pooled by weight (1% per collection interval) to generate a composite fecal sample for each study participant across 0–120 h (with the exception of participant R003, whose fecal samples were pooled over 0–192 h).

An aliquot of the pooled plasma sample underwent successive extraction with two volumes of methanol, followed by 0.5 volumes of water and two volumes of methanol. The combined extraction supernatants were evaporated to dryness under a gentle stream of nitrogen and reconstituted in a 1:1 (*v/v*) mixture of methanol and water for metabolite profiling. Reconstituted samples from 6 study participants were pooled in equal volumes for metabolite identification.

For urine samples, protein precipitation was performed by adding three volumes of acetonitrile (*v/v*) to pooled urine aliquots. For fecal samples, pooled homogenates underwent two successive extractions with two volumes of methanol per extraction. Following vortex-mixing and centrifugation, supernatants from each matrix were evaporated separately to dryness under a gentle stream of nitrogen, and the residue was reconstituted in a 1:1 (*v/v*) mixture of methanol and water for metabolite profiling. Finally, matrix-specific reconstituted extracts from 6 participants were pooled in equal volumes for metabolite identification.

Radioactive metabolite profiles were acquired through high-performance liquid chromatography coupled with low-energy radioisotope detection (TopCount-HTS solid scintillation counter and AR™ online radioisotope detector). Radiochromatograms of each sample were integrated to determine the peak area of individual radioactive peaks and their percentage contribution to the total sample radioactivity. Subsequently, the percentage of administered dose (%Dose) for JP-1366 and its metabolites in excreta (urine and feces), and the percentage of total plasma radioactive exposure (%AUC) for JP-1366 and its metabolites were calculated. Following the radioactivity-based analytical method, the metabolites %Dose and %AUC were calculated as molar equivalents of JP-1366.

Mass spectrometric signals of metabolites were acquired using an Acquity™ I-Class UPLC system (Waters Corporation, Milford, MA, United States) coupled to a Q-TOF 5600+ high-resolution mass spectrometer (SCIEX, Framingham, MA, United States). Structural characterization of metabolites was achieved by systematically comparing MS/MS fragmentation patterns with those of the parent drug. Metablism and clearance pathways of JP-1366 were developed based on structurally characterized metabolites identified in plasma, urine, and feces.

### 2.6 Safety assessment

Key safety parameters included adverse events (AEs), vital signs (blood pressure, heart rate, and body temperature), physical examinations, laboratory tests (complete blood count, urinalysis, blood biochemistry panel, coagulation profile, and stool analysis with occult blood testing) and 12-lead electrocardiographic (ECG) findings. AEs occurring post-dose were documented and graded by study investigators according to the Common Terminology Criteria for Adverse Events (CTCAE v5.0, National Cancer Institute).

### 2.7 Statistical analysis

Pharmacokinetic (PK) parameters for total radioactivity concentration in whole blood and plasma, JP-1366, and its metabolite M1 in plasma were derived via non-compartmental analysis (NCA) using Phoenix WinNonlin^®^ (version 8.3, Certara, United States). PK parameters included peak concentration (C_max_), time to peak (T_max_), area under the concentration-time curve (AUC_0-t_ and AUC  0−∞ ), elimination half-life (t_1/2_), apparent clearance (CL/F) and apparent volume of distribution (V_d_/F). Blood-to-plasma ratio (B/P ratio) was calculated as total radioactivity concentration in whole blood divided by total radioactivity concentration in plasma. Based on the individual actual administered dose, total and cumulative radioactivity recoveries in urine and/or feces were calculated using Microsoft Excel (version 2024), and the cumulative excretion rate curves were generated. Statistical analyses were performed using SAS (version 9.4). All PK parameters were calculated on an individual basis and then averaged. Continuous variables were summarized as arithmetic mean ± standard deviation (SD), and categorical variables were summarized as frequencies and percentages. In addition, geometric mean was also reported for C_max_ and AUC.

## 3 Results

### 3.1 Baseline characteristics, administered dose level and safety results

All 6 enrolled male study participants completed the study. The median age (range) of study participants was 32.5 (25–42) years. The mean (±SD) height was 170 ± 6.22 cm, and the Body Mass Index (BMI) was 23.7 ± 1.86 kg/m^2^ (range: 20.8–25.8 kg/m^2^). Ethnic composition included Han Chinese (n = 5) and Manchu (n = 1). All enrolled study participants reported no clinically relevant history of allergic diseases, substance abuse, blood donation or significant blood loss, tobacco use, or alcohol consumption within protocol-defined time window. No clinically significant abnormalities were detected in screening assessments.

The administered dose for each study participant was quantified by comparing radioactivity in dosing vials before and after administration. Following calculation, the actual administered dose of unlabeled JP-1366 (salt basis) was 20.7 ± 0.0830 mg (range: 20.6–20.8 mg), while that of [^14^C]JP-1366 was 105 ± 1.67 μCi (range: 104–108 μCi).

[^14^C]JP-1366 suspension was well-tolerated at the study dose in all subjects. 3 treatment-emergent adverse events (TEAEs) of hypertriglyceridemia occurred in 3 study participants (50%, 3/6). All TEAEs were assessed as possibly drug-related per CTCAE Grade 1 criteria, resolved spontaneously without intervention, and no serious adverse events were reported. JP-1366 demonstrated a favorable safety profile in healthy Chinese participants, consistent with a single-dose escalation study in a Korean male population that exhibited good tolerability up to 60 mg (Hwang et al., 2023).

### 3.2 Pharmacokinetics

**FIGURE 2:**
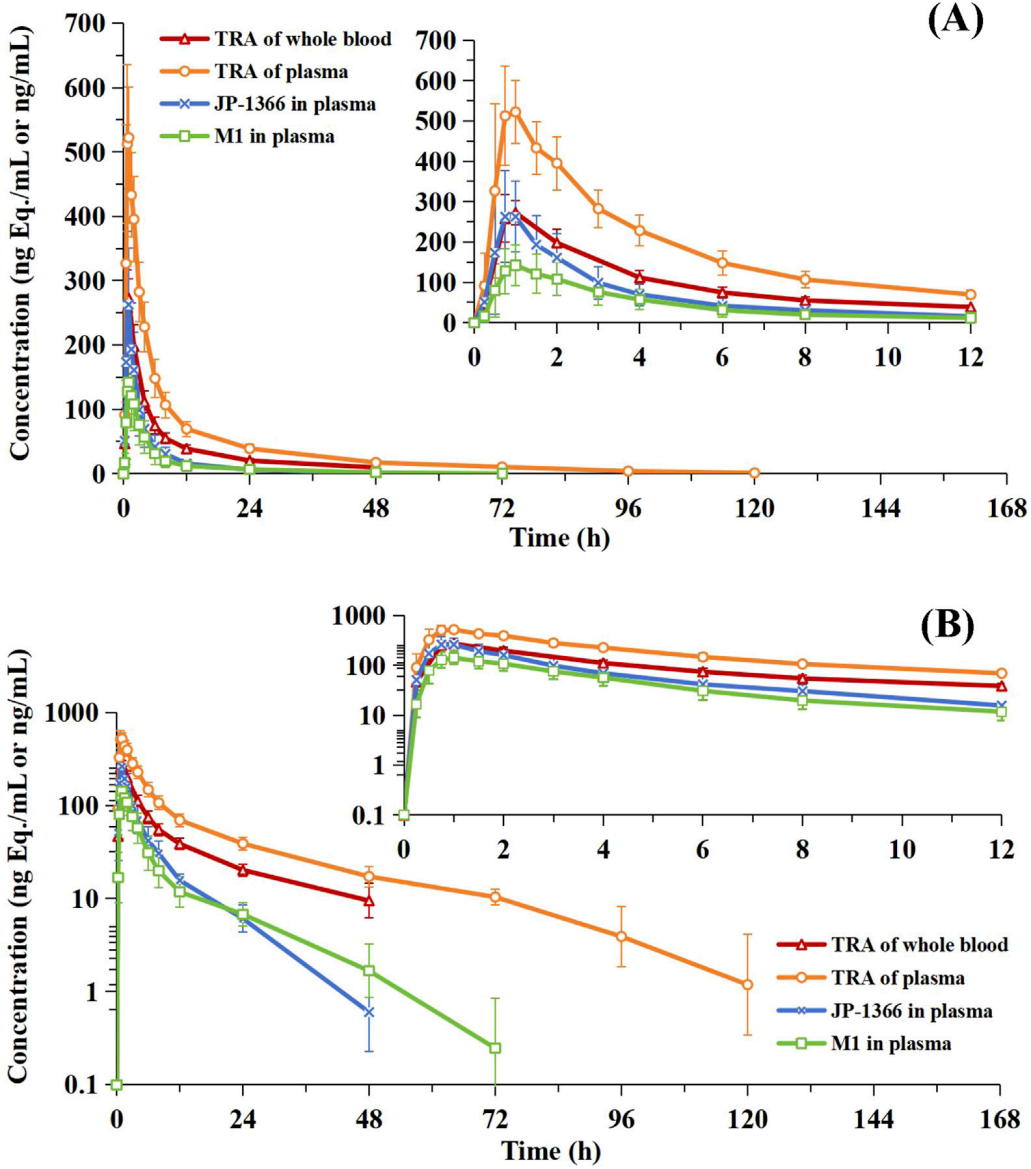
Concentration - time profiles of total radioactivity in whole blood and plasma, JP-1366, and metabolite M1 in plasma (Mean ± SD, n = 6). Panel **(A)**: linear scale; Panel **(B)**: Semi-logarithmic scale.

Figure 2 presents the mean concentration-time profiles of total radioactivity (TRA) in whole blood and plasma, JP-1366, and its metabolite M1 in plasma following a single oral administration of [^14^C]JP-1366. Corresponding pharmacokinetic parameters are summarized in Table 1.

**TABLE 1:** Pharmacokinetic parameters of total radioactivity in whole blood and plasma, JP-1366, and its metabolite M1 in plasma following a single oral dose of [^14^C]JP-1366 under fasting conditions in healthy male study participants (Mean ± SD).

PK parameters	Unit	TRA of whole blood (n = 6)	TRA of plasma (n = 6)	JP-1366 (n = 6)	M1 (n = 6)
T_max_	h	1.00 (0.750, 1.00)	0.875 (0.500, 1.00)	0.875 (0.500, 1.00)	1.00 (0.750, 1.00)
C_max_	ng Eq./mL or ng/mL^a^	306 ± 40.0	575 ± 94.6	288 ± 101	145 ± 50.4
		303 (13.8)^b^	569 (16.6)^b^	275 (32.8)^b^	138 (35.7)^b^
AUC_0-t_	h·ng Eq./mL or h·ng/mL^a^	1930 ± 339	4150 ± 572	990 ± 305	739 ± 269
		1900 (19.9)^b^	4110 (15.2)^b^	956 (28.9)^b^	688 (46.8)^b^
AUC 0−∞	h·ng Eq./mL or h·ng/mL^a^	2270 ± 363	4470 ± 591	1030 ± 304	790 ± 291
		2250 (17.7)^b^	4430 (14.6)^b^	997 (28.2)^*^	736 (46.1)^b^
t_1/2_	h	18.7 ± 5.08	27.9 ± 11.9	7.78 ± 3.08	12.6 ± 5.66
CL/F	L/h	6.12 ± 1.19	3.09 ± 0.496	14.0 ± 3.67	NC
V_d_/F	L	160 ± 29.8	122 ± 45.2	165 ± 90.3	NC

n: Number of study participants included in descriptive statistics; T_max_ recorded as median (minimum, maximum); PK, parameters that could not be calculated were denoted as “NC” in the list.

^a^ ng Eq./mL and h·ng Eq./mL refers to ng-equivalent of JP-1366.

^b^ C_max_, AUC_0-t_ and AUC  0−∞ are also reported as geometric mean (coefficient of variation, CV%).

For total radioactivity (TRA), expressed as molar equivalents of JP-1366, the median T_max_ values were 1.00 h in whole blood and 0.875 h in plasma. C_max_ was 306 ± 40.0 ng Eq./mL (whole blood) and 575 ± 94.6 ng Eq./mL (plasma). Corresponding AUC  0−∞ values were 2270 ± 363 h·ng Eq./mL (whole blood) and 4470 ± 591 h·ng Eq./mL (plasma). The estimated t_1/2_ was 18.7 h (whole blood) and 27.9 h (plasma), with V_d_/F of 160 L and 122 L, and CL/F of 6.12 L/h and 3.09 L/h, respectively.

For JP-1366 and its metabolite M1 in plasma, median T_max_ values were 0.875 h and 1.00 h, respectively. C_max_ was 288 ± 101 ng/mL for JP-1366 and 145 ± 50.4 ng/mL for M1. Corresponding AUC  0−∞ values were 1030 ± 304 h·ng/mL (JP-1366) and 790 ± 291 h·ng/mL (M1). The estimated t_1/2_ was 7.78 h for JP-1366 and 12.6 h for M1. Pharmacokinetic parameters for JP-1366 included V_d_/F of 165 L and CL/F of 14.0 L/h.

The blood-to-plasma ratio of total radioactivity (TRA) concentration was 0.544 ± 0.0638, ranging from 0.505 to 0.657 across study participants and time points. All observed values were below 1. This suggested limited distribution of drug-related material into blood cells.

### 3.3 Excretion of radioactivity in urine and feces

**FIGURE 3:**
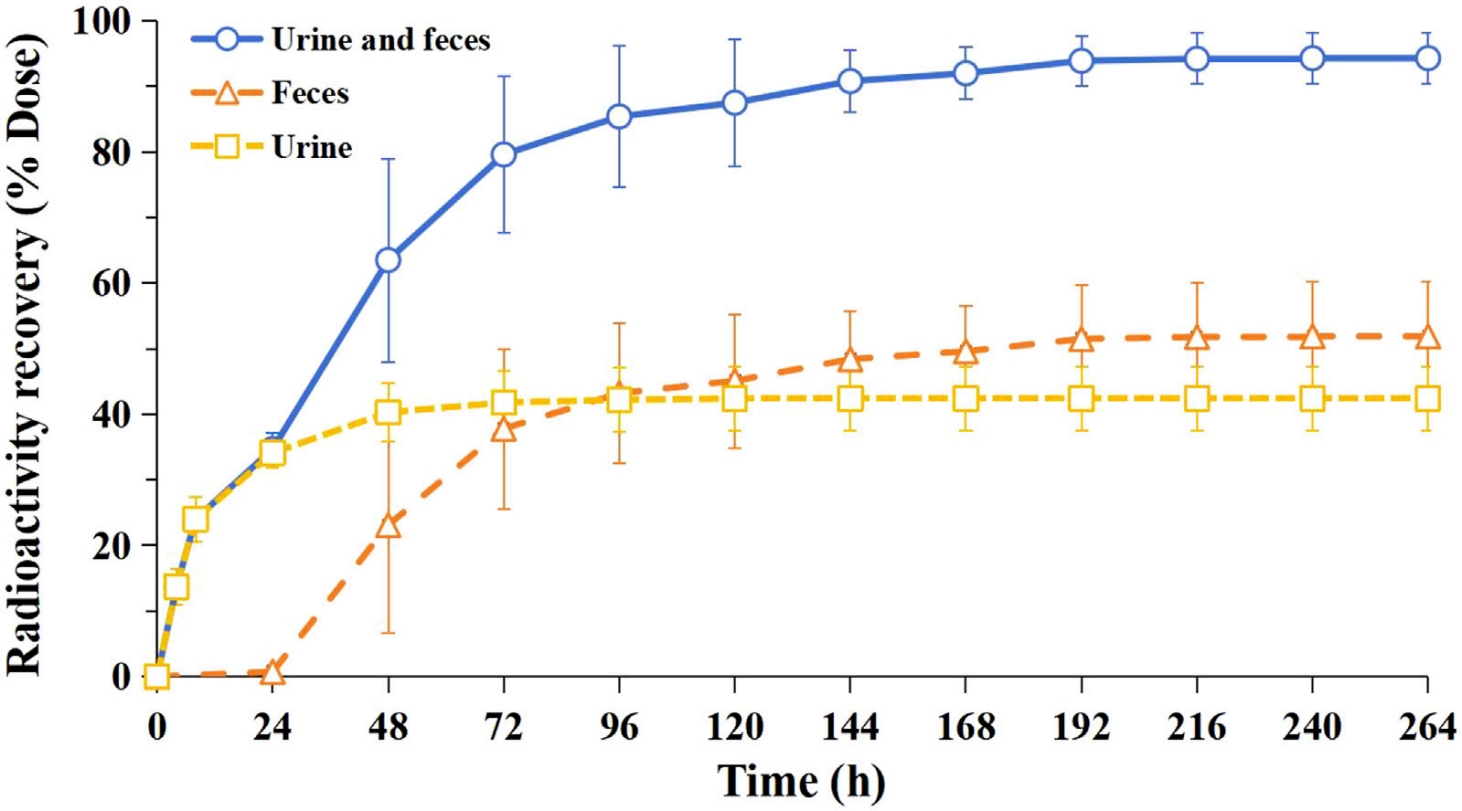
Cumulative excretion profile of radioactivity in urine and feces after a single oral dose of [^14^C]JP-1366 in healthy male study participants (mean ± SD, n = 6).

The cumulative recovery of total radioactivity in excreta over 0–264 h was 94.3% ± 3.92% of the administered dose. Fecal excretion accounted for 51.9% ± 8.27% and urinary excretion for 42.4% ± 4.92%, indicating fecal elimination as the primary elimination pathway and renal excretion as the secondary route. The majority of radioactivity (85.4% ± 10.7% of the dose) was excreted within 0–96 h, representing 90.6% of the total recovered radioactivity. The cumulative excretion rate curves are shown in Figure 3 and detailed data are listed in Table 2.

**TABLE 2:** Summary of cumulative radioactivity recovery (%) in urine and feces of 6 healthy male study participants.

Subject ID	Urine	Feces	Total (urine and feces)
R001	36.9	62.8	99.7
R002	47.8	43.1	90.9
R003	38.0	60.8	98.8
R004	39.3	52.3	91.6
R005	45.2	46.4	91.6
R006	47.4	45.9	93.2
N	6	6	6
Mean	42.4	51.9	94.3
SD	4.92	8.27	3.92

### 3.4 Metabolite profiling

**FIGURE 4:**
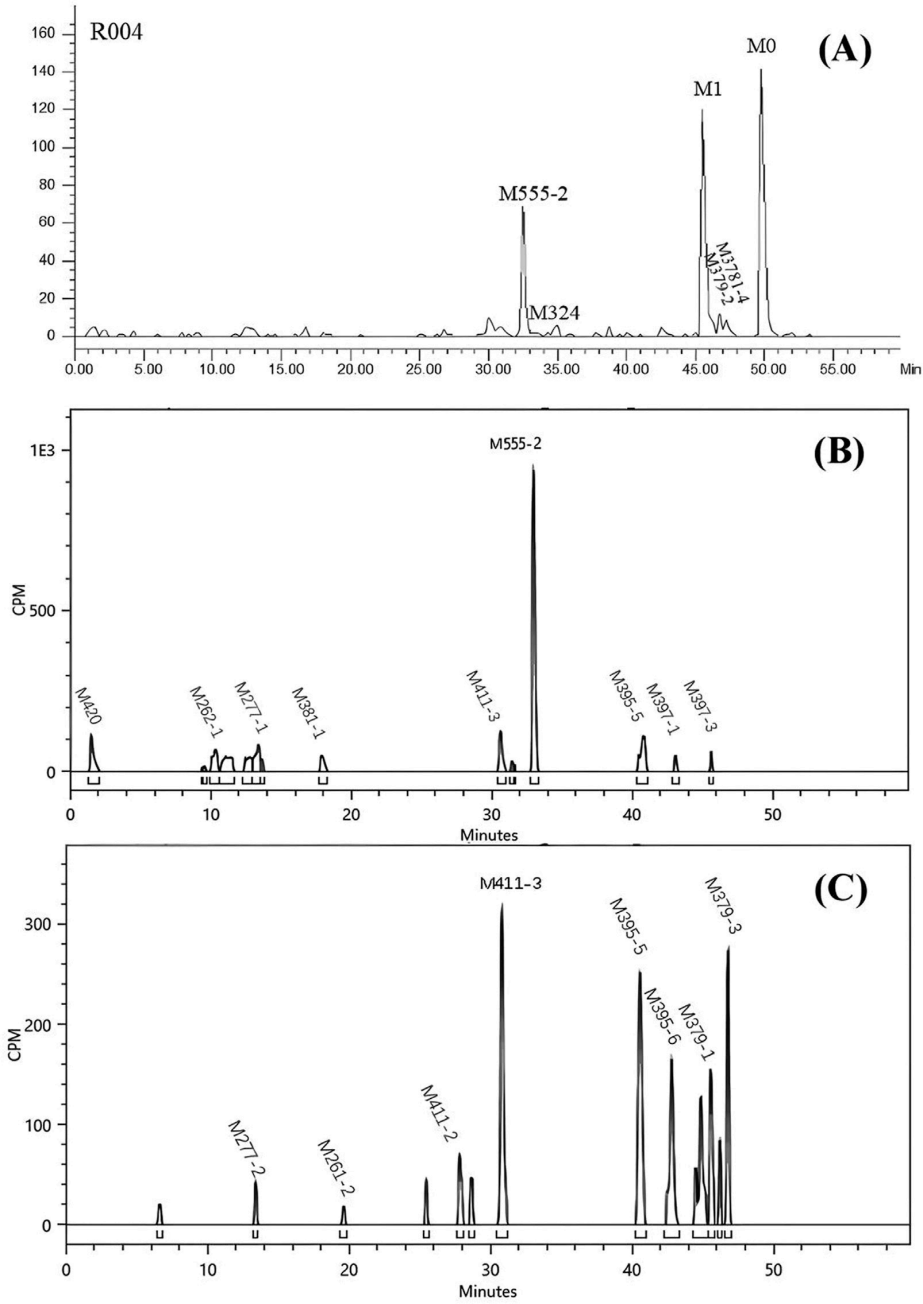
Representative radiochromatograms of metabolites in human plasma (0–48 h) **(A)**, urine (0–48 h) **(B)**, and feces (0–120 h) **(C)** following oral administration of 20 mg (100 µCi) [^14^C]JP-1366.

**FIGURE 5:**
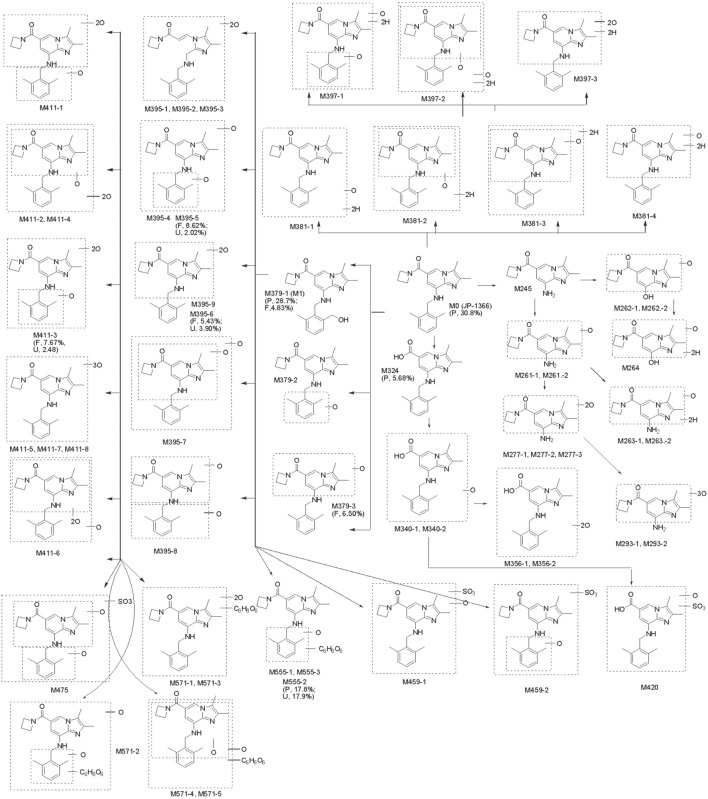
Possible metabolic pathways of JP-1366 in healthy study participants. P, F, U: plasma, feces, urine; values in parentheses represent % of plasma total radioactivity for plasma samples or % administered dose for feces/urine samples. It depicts the specific concentrations of major metabolites representing ≥5% of AUC or administered dose; those below this threshold are not listed.

JP-1366 was extensively metabolized, and 57 metabolites were identified in human with UPLC/Q TOF MS by comparing their mass fragment pathways with those of JP-1366 and M379 (M1) reference standard. The identified metabolites included 45 Phase I metabolites and 12 Phase II metabolites. The radiochromatograms of plasma, urine, and fecal sample are shown in Figure 4 and the structures and of the metabolites are presented in Figure 5.

#### 3.4.1 Plasma metabolite profiling

In pooled plasma samples, the major drug-related components were JP-1366 (M0) and the oxidative metabolite M379-1 (M1), accounting for 30.8% and 28.7% of plasma total radioactivity, respectively. Minor components included the glucuronidated oxidative metabolite M555-2 (17.8%) and the amide hydrolyzed metabolite M324 (5.68%). Other metabolites constituted less than 5% of plasma radioactivity. Unidentified drug-related material accounted for 2.37% of total radioactive component.

#### 3.4.2 Urine metabolite profiling

Urinary elimination accounted for 42.4% of the administered radioactive dose, with 40.3% excreted within 48 h. Radiochromatographic analysis revealed 30 drug-related peaks, dominated by the glucuronidated oxidative metabolite M555-2 accounting for 17.9% of administered dose. Metabolite M379-1 (M1) and other minor components each accounted for <5% of the administered dose, while parent drug was not detected.

#### 3.4.3 Fecal metabolite profiling

Fecal elimination accounted for 51.9% of the administered radioactive dose, with 47.8% excreted within 120 h (Subject R003 exhibited delayed excretion, with 59.9% of dose eliminated between 0–192 h). Radiochromatographic analysis of pooled fecal samples revealed 37 drug-related peaks. The major drug-related components were the oxidative metabolite M379-3 (6.50%), the dioxidative metabolite M395-5 (8.62%) and M395-6 (5.43%), and the trioxidative metabolite M411-3 (7.67%). Metabolite M379-1 (M1) and other minor components each constituted less than 5% of the administered dose. No radioactive peak corresponding to the parent drug was detected, indicating minimal intact drug excretion in feces.

#### 3.4.4 Major metabolic pathways

Metabolite identification results for JP-1366 suggest that its main metabolic pathways in healthy male subjects involve oxidations (Phase I) and the following glucuronidation (Phase I/II). The major metabolic pathways of the proposed metabolites of JP-1366 in humans are summarized in Figure 5; Table 3.

**TABLE 3:** Metabolic pathways and metabolites of JP-1366 identified in healthy male study participants.

Metabolic pathway	Metabolite ID
Parent drug	M0
N-Dealkylation	M245
N-Dealkylation and oxidation	M261–1, M261-2
Oxidative deamination and oxidation	M262–1, M262-2
N-Dealkylation, oxidation and hydrogenation	M263–1, M263-2
Oxidative deamination, oxidation and hydrogenation	M264
N-Dealkylation and dioxidation	M277–1, M277–2, M277-3
N-Dealkylation and trioxidation	M293–1, M293-2
Amide hydrolysis	M324
Amide hydrolysis and oxidation	M340–1, M340-2
Amide hydrolysis and dioxidation	M356–1, M356-2
Oxidation	M379-1 (M1), M379–2, M379-3
Oxidation and hydrogenation	M381–1, M381–2, M381–3, M381-4
Dioxidation	M395–1, M395–2, M395–3, M395–4, M395–5, M395–6, M395–7, M395–8, M395-9
Dioxidation and hydrogenation	M397–1, M397–2, M397-3
Trioxidation	M411–1, M411–2, M411–3, M411–4, M411–5, M411–6, M411–7, M411-8
Amide hydrolysis, oxidation and sulfation	M420
Oxidation and sulfation	M459–1, M459-2
Dioxidation and sulfation	M475
Oxidation and glucuronidation	M555–1, M555–2, M555-3
Dioxidation and glucuronidation	M571–1, M571–2, M571–3, M571–4, M571-5

## 4 Discussion

JP-1366 is a novel potassium-competitive acid blocker (P-CAB) that achieves rapid, potent, and sustained gastric acid suppression. Its optimized pharmacokinetic profile supports once-daily dosing for acid-related disorders such as gastroesophageal reflux disease and peptic ulcer bleeding. This mass balance study was conducted to comprehensively characterize the absorption, distribution, metabolism and excretion (ADME) profile of JP-1366, thereby informing rational clinical dosing regimens. With a single 20 mg dose, the PK profile of JP-1366 in Chinese participants was comparable to that in healthy Korean participants, with consistent key parameters (C_max_: 207 ng/mL; AUC  0−∞ : 883 ng·h/mL; t_1/2_: 8.63 h) (Hwang et al., 2023). The similar exposure levels further support the representativeness of the mass balance data obtained in this study.

Following oral administration, JP-1366 was rapidly absorbed, with median T_max_ values of 0.875 h observed for both the parent drug and total radioactivity in human plasma. Based on parameter of C_max_, AUC_0-t_, and AUC  0−∞ , JP-1366 accounted for 49.1%, 23.7%, and 23.0% of total plasma radioactivity exposure, respectively. Corresponding contributions of metabolite M1 on a molar basis were 24.0%, 16.7%, and 16.6%. Differential clearance kinetics were observed: rapid elimination for JP-1366 (t_1/2_ = 7.78 h), intermediate for M1 (t_1/2_ = 12.6 h), and slow for total radioactivity (t_1/2_ = 27.9 h). Collectively, these data indicate the presence of slowly eliminated metabolites beyond JP-1366 and M1 in systemic circulation. The whole blood-to-plasma concentration ratio of total radioactivity (0.505–0.657) indicated negligible binding of drug-related components to blood cells.

Fecal excretion was the predominant elimination pathway for drug-related material, accounting for 51.9% of the administered dose, whereas renal excretion in urine represented a secondary route, contributing 42.4%. In SD rats, excretion studies demonstrated that biliary-fecal elimination was the primary route for [^14^C]JP-1366, with recovery rates of 81.7% in feces and 15.3% in urine. Consistent primary excretion pathways were observed across species, with fecal elimination being predominant in both humans and rats. Nevertheless, the proportion of drug excreted via the renal route was significantly higher in humans (42.4%) than in rats (15.3%). These results share a comparable excretion profile with other P-CABs like Tegoprazan, which was eliminated in both urine (50.51% ± 3.35%) and feces (47.26% ± 3.06%) (Bian et al., 2025).

Plasma metabolite profiling demonstrated that, in addition to the parent drug (30.8% of total radioactivity exposure), metabolites exceeding 10% of plasma exposure (%AUC) were M1 (28.7%) and M555-2 (17.8%). Among these, M1 was a known monooxidative metabolite that has been quantitatively analyzed. It confirmed that the exposure of M1 in rats (under no observed adverse effect level) could adequately cover the exposure observed in humans at clinical dose level. The other major metabolite, M555-2, was a Phase II metabolite formed via mono-oxidation followed by glucuronidation. Phase II glucuronidation typically enhances hydrophilicity and leads to the loss of pharmacologic activity, which are generally devoid of toxicological concern (International Council for Harmonisation of Technical Requirements for Pharmaceuticals for Human Use, 2013). Therefore, no further nonclinical safety evaluation was required for these high-abundance metabolites.

*In vivo* biotransformation was extensive, with oxidations (Phase I) and the following glucuronidation (Phase I/II) constituting the principal metabolic routes. Consequently, almost all of the administered dose was ultimately eliminated as metabolites through fecal and urinary pathways. A^14^C-labeled mass balance study on vonoprazan, another P-CAB drug, also demonstrated its extensive *in vivo* metabolism, with 67.4% and 31.1% of the administered radioactivity recovered in urine and feces, respectively. Specifically, metabolites accounted for 59% of the total dose in urine and 29% in feces (Food and Drug Administration, 2023). For JP-1366, It is worth noting that both the parent drug and pharmacologically active metabolite M1 demonstrated minimal renal elimination. The major urinary metabolite M555-2 was pharmacologically inactive, with a fractional excretion ratio of 17.9%. These data will support the regulatory assessment, particularly for patients with renal impairment. (Food and Drug Administration, 2024).

Given that neither JP-1366 nor its metabolite M1 exhibited time-dependent pharmacokinetic characteristics in previous studies, a single-dose study design was adopted. Only six male participants were enrolled and dosed due to considerations of the potential radiation risk and reproductive toxicity. In addition, the participants enrolled in this study (aged 25–42) were younger than those in most patient with GERD. Therefore, these limitations may affect the generalizability of the present study.

In conclusion, a mass balance study using ^14^C-labeled JP-1366 was conducted in healthy Chinese male adults. The mean cumulative recovery of radioactivity reached 94.3% within 264 h, with fecal excretion accounting for 51.9% and urinary excretion for 42.4%. JP-1366 was extensively metabolized *in vivo* and was ultimately eliminated primarily as metabolites via urine and feces. The principal metabolic pathways involved oxidations (Phase I) and the following glucuronidation (Phase I/II). Furthermore, the drug was well tolerated and demonstrated a favorable safety profile in the study participants.

## Data Availability

The raw data supporting the conclusions of this article will be made available by the authors, without undue reservation.
